# Complex Interplay Between Obesity and BRCA1/2‐Associated Breast Cancer: An Overview

**DOI:** 10.1111/obr.13969

**Published:** 2025-06-16

**Authors:** Cinzia Giordano, Marianna Puzzo, Rocco Malivindi, Debora Cristofaro, Luca Gelsomino, Daniela Bonofiglio, Carlo Capalbo, Sebastiano Andò, Ines Barone, Stefania Catalano

**Affiliations:** ^1^ Department of Pharmacy, Health and Nutritional Sciences University of Calabria Cosenza Italy; ^2^ Centro Sanitario, University of Calabria Cosenza Italy; ^3^ Clinical Laboratory Unit A.O. “Annunziata” Cosenza Italy; ^4^ Oncology Unit A.O. “Annunziata” Cosenza Italy

**Keywords:** BRCA1, BRCA2, breast cancer, obesity

## Abstract

The steadily increasing prevalence of obesity and its association with a growing number of malignancies, including breast cancer, has made this disease spectrum an urgent and critical public health priority, demanding immediate attention and comprehensive action. To date, a growing body of research has been dedicated to the study of obesity/breast cancer biological link, with the most well‐documented mechanisms involving chronic inflammation, altered adipokine levels, dysregulated hormone signaling, and insulin/growth factor pathways. Despite significant progress, a substantial gap persists in our present comprehension of the association between adiposity and breast cancer biology in individuals with mutations in the *BRCA1/2* genes, the most widely known high‐penetrance genes involved in severe breast cancer risk. In this review, we first give an overview of the contribution of *BRCA1/2* gene mutations in breast cancer development. Then, we discuss the emerging mechanistic evidence linking obesity with breast cancer, highlighting the impact of metabolic and hormonal factors in *BRCA* mutation carriers. Insights into the cross‐talk between obesity and breast cancer development in *BRCA* mutation carriers may pave the way to improve proper personalized clinical management of BRCA1/2‐associated breast cancer.

**Abbreviations**
ABCSG‐6a: Austrian Breast and Colorectal Cancer Study Group Trial 6a
Abraxas: BRCA1 A Complex Subunit
AdipoR1: adiponectin receptor 1
AdipoR2: adiponectin receptor 2
AMPK: AMP‐activated protein kinase
APPL1: adaptor protein, phosphotyrosine interacting with PH domain and leucine zipper 1
ATAC: arimidex, tamoxifen alone or in combination
ATM: ataxia‐telangiectasia mutated
ATR: ATM‐ and Rad3‐related
BARD1: BRCA1‐associated RING domain protein 1
BC: breast cancer
BMI: body mass index
*BRCA1*
: breast cancer gene 1
*BRCA2*
: breast cancer gene 2
BRCT: BRCA1 C‐terminal domain
BRIP1: BRCA1 interacting protein C‐terminal helicase 1
CCL2: chemokine (C‐C motif) ligand 2
c‐Src: proto‐oncogene tyrosine kinase c‐SRC
CtIP: C‐terminal binding protein 1 (CtBP1) interacting protein
CYP19: aromatase cytochrome P450
CYP1A1: cytochrome P450 family 1 subfamily A member 1
DMC1: DNA meiotic recombinase 1
DSBs: double‐strand breaks
EGFR: epidermal growth factor receptor
ER: estrogen receptor
EVs: extracellular vesicles
FANCD2: Fanconi anemia complementation group D2
H2AX: H2A histone family member X
HER2: human epidermal growth factor receptor 2
HIF‐1α: hypoxia inducible factor‐1α
HR: homologous recombination
Hsp90: heat shock protein 90
IGF‐IR: insulin‐like growth factor‐1 receptor
IL: interleukin
LKB1: liver kinase B1
MAPK: mitogen‐activated protein kinase
MHC: major histocompatibility complex
miRNAs: microRNAs
MRN: MRE11–RAD50–NBS1 complex
NCCN: National Comprehensive Cancer Network
NF‐κB: nuclear factor‐κB
NHEJ: nonhomologous end joining
NLRC4: NOD‐like receptor CARD domain containing 4
NLS: nuclear localization signals
Ob: obese
OR: odds ratio
PhePP: phenylalanine‐proline‐proline
PI3K: phosphatidylinositol 3‐kinase
PR: progesterone receptor
PVs: pathogenic variants
Rab GTPases: Ras‐associated binding proteins
RAD51: radiation sensitive protein 51 recombinase
RAP80: receptor‐associated protein 80
TNBC: triple negative breast cancer
TNF‐α: tumor necrosis factor α
TRAIL: tumor necrosis factor related apoptosis‐inducing ligand
Tsg101: tumor susceptibility gene 101
VEGF: vascular endothelial growth factor

## 1 Introduction

The continuous global rise in overweight and obesity prevalence is becoming one of the most serious public health problems with very significant clinical implications, because obesity represents a well‐recognized modifiable risk factor for several noncommunicable diseases, including breast cancer (BC). BC is the most commonly occurring malignancy and the first leading cause of death among women worldwide [1]. BC onset, development, and progression are profoundly influenced by molecular messages coming from the adipose tissue characterized, in obesity condition, by metabolic and inflammatory changes able to modify physiological homeostasis (e.g., imbalance in adipokine secretion, increase in estrogen and insulin signaling, release of mediators of chronic inflammation) [2].

Approximately 5%–10% of BC cases are ascribed to germline pathogenic variants (PVs) in BC susceptibility genes, with *BRCA1* and *BRCA2* mutations being the most prominent and well‐studied genetic contributors [3]. The primary function of BRCA1/2 is mainly linked to the maintenance of genome stability through involvement in DNA repair processes including homologous recombination, protection of the DNA replication fork, regulation of transcription, and DNA damage response pathways. Indeed, the loss of BRCA functions leads to genomic instability, which ultimately drives the oncogenic transformation of nontumorigenic cells into tumor‐initiating cells or cancer stem cells, fuelling tumor progression [4].

It has been estimated, from a prospective cohort of 9856 mutation carriers, that the cumulative BC risk to age 80 years was 72% and 69% for *BRCA1* and *BRCA2* carriers, respectively, compared with 12% in the entire population [5]. In women with inherited *BRCA1*/*BRCA2* mutations BC incidence increased rapidly in early adulthood (ages 30 to 40 years) for *BRCA1* and (ages 40 to 50 years) for *BRCA2* carriers, then continued at a comparable incidence until age 80 years old [5]. However, the incomplete penetrance of these inherited mutations suggests that modifiable environmental risk factors as life style, excessive body weight, and physical inactivity may influence BC risk in this cohort of subjects [6]. Thus, to improve clinical management of *BRCA1* and *BRCA2* mutation carriers is necessary a deep knowledge concerning the impact of modifiable factors, as obesity, on BC risk, that may provide the oncologists additional recommendations for the care of patients with BC.

This review aims to shed light on the intricate and partially elucidated relationship between obesity and BRCA‐associated BC, also, focusing on the molecular mechanisms that facilitate this deleterious association. First, the role of *BRCA1* and *BRCA2* gene mutations in BC development will be discussed. Then, we will address the molecular players by which obesity‐associated features may affect breast malignancy. Finally, the impact of obesity in *BRCA 1* and *BRCA 2* mutation carriers will be highlighted.

## 2 BRCA1/2 and Breast Malignancies

*BRCA1* and *BRCA2* are tumor suppressor genes that encode large, ubiquitous and multifunctional proteins of 1863 and 3418 amino acids respectively [7, 8] that play a crucial role in DNA repair, cell‐cycle control and chromosomal stability [9]. *BRCA1* and *BRCA2* pathogenic variants (PVs) are present in approximately 0.1%–0.2% of the general population [10]. PVs in *BRCA1* and *BRCA2* genes are associated with about 3%–8% of all cases and 15%–20% of familial cases of BC [11]. Genetic alterations in the BRCA1 gene represent the primary cause of hereditary breast cancer, accounting for approximately 40%–45% of cases. [12, 13] By the age of 70, it has been estimated that the cumulative risk for developing BC is 65%–80% and 45%–85% for *BRCA1* and *BRCA2* mutation carriers, respectively [14]. There are more than 1600 and 1800 known variants in *BRCA1* and *BRCA2*, respectively, the majority of these are responsible for frameshifts, which in turn result in the production of missense or nonfunctional proteins [15]. In addition to BC, PVs in *BRCA1* gene increase the risks of ovarian cancer in women and prostate cancer in men, while *BRCA2* PVs are associated with an increased risk of cholangiocarcinoma, gastric cancer, and melanoma [16, 17].

### 2.1 BRCA Genes: Structure and Function

The *BRCA1* and *BRCA2* genes are located on chromosomes 17q21 and 13q12.3, respectively. The *BRCA1* gene consists of 24 coding exons, with exon 11 being the largest, encoding over 60% of the BRCA1 protein [7] while *BRCA2* gene comprises 27 exons, with exons 10 and 11 frequently being sites of pathogenic mutations that result in insertions or deletions, leading to premature stop codons or missense mutations [8]. Both BRCA1 and BRCA2 proteins contain different functional domains. Particularly, exons 2–5 of *BRCA1* gene encode the N‐terminal zinc‐binding RING finger domain (amino acids #10‐109), a critical region involved in protein–protein interactions and exhibiting E3 ubiquitin ligase activity. This domain forms a stable heterodimer with the BRCA1‐associated RING domain protein 1 (BARD1), which is essential for the DNA damage response through its role in ubiquitination [18]. Exons 15–23 encode the BRCA1 C‐terminal (BRCT) domain (amino acids #1640‐1729 and #1760‐1821) [19] a conserved structure crucial for interacting with phosphorylated proteins such as CtIP (C‐terminal binding protein interacting protein), BRIP1 (BRCA1 interacting protein C‐terminal helicase 1) and Abraxas (BRCA1 A Complex Subunit), which are involved in the DNA damage response [20, 21]. Exons 11–13 codify for the central part of BRCA1 proteinthat includes two nuclear localization signals (NLS), a coiled‐coil domain (amino acids #1367‐1437), and a serine‐glutamine (SQ) cluster (amino acids #1280‐1524), which contains serine and threonine residues phosphorylated by ATM and ATR, further enhancing its role in DNA repair and cell cycle regulation [22]. Mutations in these regions are often identified in BC patients.

**FIGURE 1:**
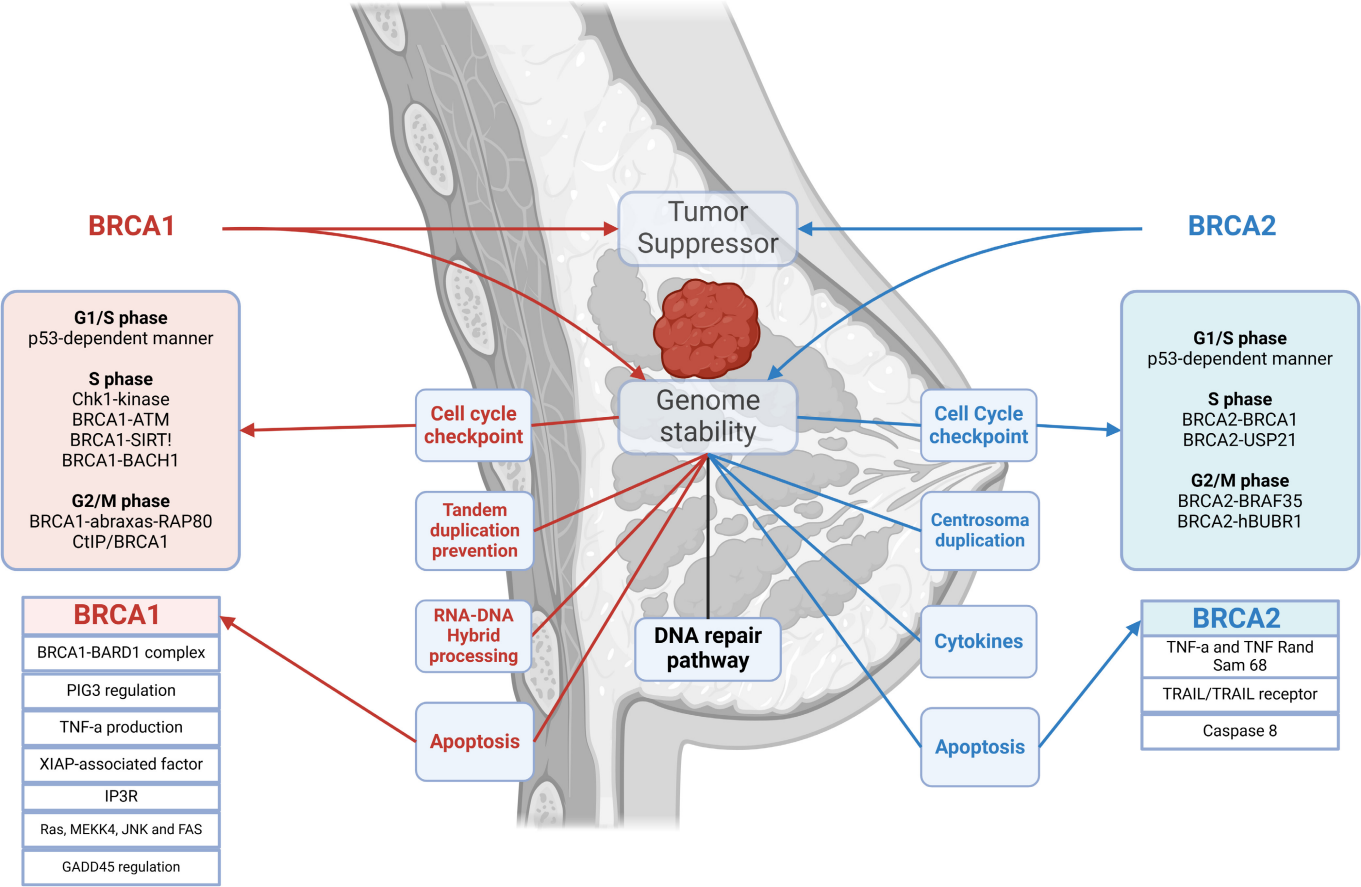
Schematic representation of the BRCA1 and 2 activities in breast cancer. *BRCA1* and *2* tumor suppressor genes play a crucial role in maintaining genomic stability, DNA repair pathways, chromatin structure regulation, transcriptional regulation, cell cycle control and apoptosis pathway. ATM, ataxia‐telangiectasia mutation; BACH1, BRCA1‐associated helicase 1; BARD1, BRCA1 associated RING domain 1; BRAF35, BRCA2‐associated factor 35; Chk1, Checkpoint kinase; CtIP, C‐terminal binding protein interacting protein; FAS, fatty acid synthase; GADD45, growth arrest and DNA damage‐inducible protein; hBUBR1, human mitotic checkpoint serine/threonine‐protein kinase BUB1 beta; IP3R, inositol 1,4,5‐trisphosphate receptor; JNK, c‐Jun N‐terminal kinase; MEKK4, mitogen‐activated protein kinase kinase kinase 4; PIG3, p53‐inducible gene 3; RAP80, receptor‐associated protein 80; RAS, rat sarcoma virus; SIRT1, sirtuin 1; TNF‐α, tumor necrosis factor alpha; TRAIL, tumor‐necrosis‐factor‐related apoptosis‐inducing ligand; USP21, ubiquitin specific peptidase 21; XIAP, X‐linked inhibitor of apoptosis protein. Created in https://BioRender.com.

The main active structure of BRCA2 protein is characterized by an α‐helical domain, three oligonucleotide/oligosaccharide‐binding folds (OB‐folds) responsible for binding single‐stranded DNA, and a C‐terminal RAD51 interaction domain (encoded by exon 27), which is critical for double‐strand DNA breaks repair [8]. The OB‐folds ensure a high affinity for both single‐stranded and double‐stranded DNA, while the RAD51 interaction domain facilitates DNA repair. Mutations that disrupt these domains, such as frameshift mutations in exon 27, are associated with an increasein cancer risk [23]. Additionally, the phenylalanine‐proline‐proline (PhePP) motif in the C‐terminal region (amino acids #2386‐2411) interacts with proteins like DMC1 and FANCD2 during meiosis, further underscoring BRCA2's role in genomic integrity [24]. The C terminus of BRCA2 contains two NLS motifs, which are crucial for its translocation into the nucleus. Several experimental and clinical data highlighted the involvement of BRCA genes a multitude of pivotal biological processes, such as DNA damage repair, cell cycle checkpoints, chromatin remodeling, and apoptosis cascades. Both BRCA1 and BRCA2 play pivotal roles in the repair of double‐strand breaks (DSBs) in DNA, which is crucial for preventing genomic instability and cancer development. BRCA1 and BRCA2 are involved in homologous recombination (HR), a precise mechanism for repairing DSBs. In response to DNA damage, BRCA1 is recruited to damage sites through the formation of a complex with Abraxas and RAP80, which facilitates the ubiquitination of histones at DSBs [25]. This process is dependent on the phosphorylation of histone H2AX and involves the ATM kinase and various other proteins [26]. Subsequently, BRCA1 promotes DNA end resection, a critical step in HR, by interacting with CtIP and the MRN complex [27, 28]. Similarly, BRCA2 directly facilitates HR by interacting with RAD51, a protein essential for strand invasion and exchange during repair [29]. BRCA2 stabilizes RAD51 nucleofilament on single‐stranded DNA, particularly after nucleotide depletion or DNA damage [30]. Mutations affecting the interaction between BRCA2 and RAD51, such as those in the BRC repeats or the C‐terminal region of BRCA2, lead to impaired HR and increased cancer susceptibility [31]. While both BRCA1 and BRCA2 are crucial for HR, only BRCA1 is suggested to have a role in nonhomologous end joining (NHEJ), another DSB repair pathway. The involvement of BRCA2 in NHEJ is less evident, with several studies indicating it does not significantly participate in this repair mechanism [32]. BRCA1 and BRCA2 also regulate cell cycle checkpoints, which are crucial for maintaining genomic integrity in response to DNA damage. BRCA1 is implicated in the regulation of checkpoints across multiple phases of the cell cycle, including G1/S, S, and G2/M phases [33, 34, 35]. BRCA1 aids in the activation of these checkpoints by modulating key proteins and signaling pathways, ensuring cells do not proceed through the cell cycle with damaged DNA [33, 36, 37]. In contrast, the role of BRCA2 in cell cycle checkpoint control is less pronounced and primarily relates to its involvement in HR. However, BRCA2 is critical for maintaining the spindle assembly and mitotic checkpoints, particularly in the G2/M transition phase [38]. BRCA1 and BRCA2 also influence apoptosis. BRCA1 is involved in various apoptotic pathways, such as those mediated by p53 and TNF‐α [39, 40]. It can modulate apoptosis through different mechanisms, including interacting with other proteins to regulate calcium release from the endoplasmic reticulum and activating stress response pathways [41, 42]. The role of BRCA2 in apoptosis is less well‐defined, but studies suggest that it may influence cell death pathways indirectly, possibly through interactions with TNF and TRAIL receptors [43, 44]. A summary of the main BRCA1 and 2 functions in BC is reported in Figure 1.

## 3 Obesity and Breast Cancer

### 3.1 Epidemiological Evidences

Overweight and obesity, defined as body mass index (BMI) ≥ 25 kg/m^2^ and BMI ≥ 30 kg/m^2^ respectively, represent serious public health problems worldwide. The World Obesity Atlas 2023 estimates that over 4 billion people may be affected by overweight and obesity resulting in an incidence over 50% of the world's population in 2035 [45]. These metabolic conditions are known to be associated with higher risk of developing different kind of cancers including those of the breast [46]. The associations of overweight and obesity with risk of invasive BC have been proved in postmenopausal women (Women's Health Initiative clinical trials). The authors found that women with overweight/obesity had an increased invasive BC risk compared with women with normal weight, and elevated BMI (≥ 35) resulted associated with risk for estrogen/progesterone receptor‐positive (ER+/PR+) BCs. In addition, higher grades of obesity were also associated with advanced disease, characterized by larger tumor size, positive lymph nodes, regional and/or distant stage, and with increased BC mortality [47]. The study from Premenopausal Breast Cancer Collaborative Group revealed an inverse linear associations of BMI with BC risk among the 758.592 premenopausal women (median age, 40.6 years) included in the study [48]. A population‐based, case–control study of 1021 women, ages 20 to 44 years, with BC (779 ER+, 182 triple negative BC −TNBC, and 60 ER−/human epidermal growth factor receptor 2‐HER2‐overexpressing) demonstrated a slight positive association of increase in BMI since age 18 years, with an increased risk of TNBC [49]. Moreover, it has been reported that in premenopausal women increasing body size was more strongly associated with luminal B (ER+/PR−) and with the more aggressive TNBC molecular subtype of tumors [50]. Regardless of menopausal status obesity is associated with reduced time‐to recurrence and increased mortality rate [51, 52]. Long‐term follow‐up of (from 1982 to 1998) of 495.477 patients with BC, reported a positive association between BMI level and disease mortality, with a more than two‐fold higher risk of mortality for women with a BMI > 40 kg/m^2^ [51]. The positive association of obesity with poorer BC survival and a higher risk of mortality was also confirmed in a systematic literature review and meta‐analysis of 82 follow‐up studies of BC survivors [52]. Patients with obesity are at increased risk for complications with anesthesia procedures, and patients with obesity undergone breast‐conserving surgery or lumpectomy had significantly higher risk of local recurrence [53, 54]. The population of patients with obesity demonstrated a significantly diminished response to systemic chemotherapy and/or endocrine therapy, even among women who received appropriate doses of drugs [55], and women with BC with elevated BMI are more likely to acquire resistance to these therapies [56]. Results from the ATAC (arimidex, tamoxifen alone or in combination) trial and analysis of the randomized ABCSG‐6a trial, revealed that treatment with aromatase inhibitors anastrozole or letrozole showed reduced treatment efficacy in women with ER+ BC with higher BMI compared with women with a healthy range BMI [54, 57].

### 3.2 Molecular Mechanism Insights

**FIGURE 2:**
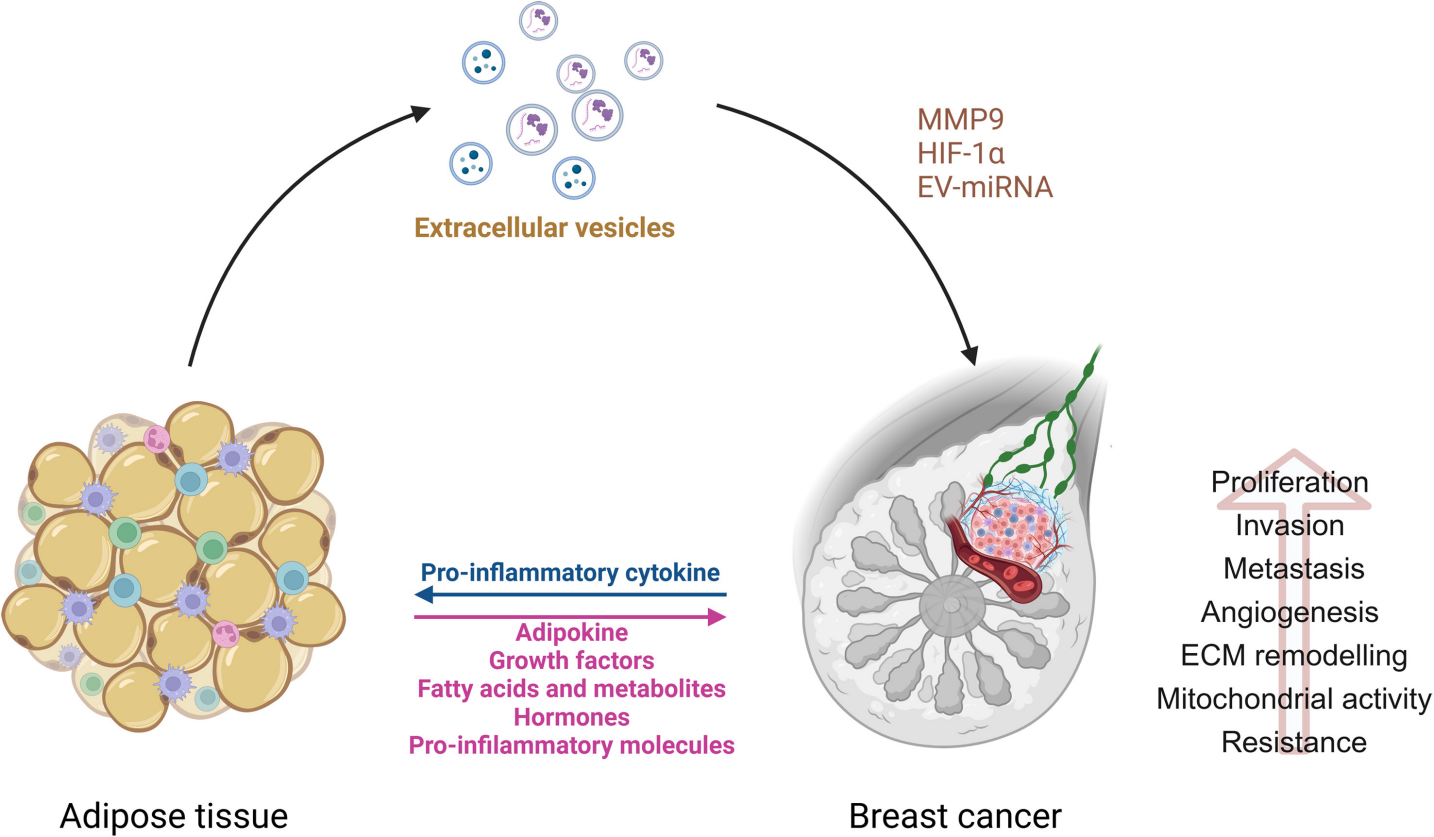
Schematic representation of the adipose tissue‐tumor cell interactions in breast cancer. In obese condition, white adipocytes undergo hypertrophy and hyperplasia resulting in several pathophysiologic changes characterized by altered secretion of different bioactive molecules as adipokines, growth factors, fatty acids and metabolites, hormones, and pro‐inflammatory molecules that support the growth, motility, epithelial to mesenchymal transition, drug resistance and metastasis outgrowth. This occurs by influencing both the phenotype of the epithelial tumor cells as well as those of the various components of the tumor microenvironment. Factor secreted by breast cancer cells profoundly influence adipocyte biology in tumor microenvironment inducing the cancer associated adipocyte phenotype. In addition to soluble factors, extracellular vesicles (EVs) released by adipocytes affect breast cancer cell biology by increasing activity of molecules as metalloproteinase 9 (MMP9) and hypoxia inducible factor‐1α (HIF‐1α) able to contribute to the metastatic process. In obesity condition, also, the EV‐miRNA signature is modified and may affect cancer behavior. Created in https://BioRender.com.

Patients with obesity affected by BC present a unique and aggressive biology that is dependent on multiple molecular mechanisms related to an environment metabolically activated by adipose tissue. In obesity, adipose tissue is characterized by hypertrophy and hyperplasia of white adipocyte cells, which results in key local and systemic changes such as increased levels of free fatty acids, triglycerides, blood glucose, insulin resistance, as well as altered secretion of growth hormones, inflammatory cytokines, and adipokines [58]. The complex molecular communication among the numerous obesity‐related factors might exert influence on diverse hallmarks of BC, either through the direct impact on the phenotype of cancer epithelial cells or indirectly by reprogramming the biology of the tumor microenvironment components [59] (Figure 2).

#### 3.2.1 Adipokines, Hormones, and Inflammatory Mediators

The expansion of fat mass in obesity is associated with an altered production of adipokines, a large group of soluble factors representing key mediators in the cross‐talk between adipocyte and BC cells. A plethora of different adipokines has been identified, and among them, the well‐characterized in the context of BC are adiponectin and leptin.

Adiponectin is an insulin‐sensitizing hormone produced by adipocytes for the maintenance of metabolic homeostasis, and its levels are decreased in obesity. Adiponectin has been shown to possess properties that include anti‐inflammatory, anti‐atherogenic, anti‐angiogenic, and anti‐diabetic effects [60]. The classical adiponectin receptors (AdipoR1) activate AMP‐activated protein kinase (AMPK), an important checkpoint of the cellular metabolic rate [61]. Treatment of BC cells with adiponectin inhibits cell adhesion, invasion, and migration, stimulates AMPK phosphorylation and activity, and reduces mTOR activity. Adiponectin enhances levels of the tumor suppressor LKB1, whose overexpression further enhances AMPK activation. LKB1 is crucial for the modulation of the adiponectin‐mediated AMPK‐S6K axis, contributing to the inhibition of malignant properties in breast carcinoma cells [62]. Many clinical studies have indicated that low adiponectin concentrations are correlated with an increased risk of BC, with an Odds Ratio (OR) of 3.63 in some cases [63] and 0.84 in others [64]. It has been demonstrated that adiponectin inhibits the growth of ER− BC cells by increasing the expression of pro‐apoptotic proteins and reducing cyclin D1 levels. However, in ER+ cells, adiponectin can stimulate cell growth by activating mitogen‐activated protein kinase (MAPK) via the AdipoR1 receptor and the adaptor protein APPL1, interacting with ERα, c‐Src, and IGF‐IR. Thus, as reported by Mauro et al., adiponectin has antiproliferative effects on ER− breast carcinoma cells but stimulates proliferation in ER+ ones [65].

On the other hand, the appetite controller hormone leptin, a molecule encoded by the Ob gene whose circulating levels are elevated in individuals with obesity, has been demonstrated to play a significant role in BC biology. It has been reported that increased leptin induces the expression of VEGF in breast carcinoma cells through HIF‐1α and NF‐κB [66]. Additionally, leptin induces the transactivation of HER‐2 and, in TNBC cells, interacts with IGF‐1 to transactivate the epidermal growth factor receptor (EGFR), thereby promoting invasion and migration in BC cells [67]. Leptin also influences the growth of ER+ BC cells through a dual mechanism involving an increase in aromatase gene expression and by a direct transactivation of ER [68, 69]. Several studies have highlighted an interaction between leptin and various members of the growth factor family [70, 71, 72]. For instance, it has been shown that leptin can induce the expression of heat shock protein 90 (Hsp90), which in turn increases the levels of HER2. This increase in HER2 levels results in reduced sensitivity of BC cells to tamoxifen treatment [73]. Obesity is, also, associated with increased serum insulin levels. It has been shown that insulin stimulates cell proliferation in normal mammary tissue and human BC cell lines by binding and signaling through insulin and Insulin‐like growth factor‐1 (IGF‐1) receptors [74]. Furthermore, insulin can increase the expression of vascular endothelial growth factor (VEGF), a potent angiogenic agent [75]. Moreover, insulin influences BC risk by influencing the levels of estrogens. Indeed, chronic hyperinsulinemia has the capacity to induce an increase in ovarian estrogen production, a reduction in hepatic secretion of sex hormone‐binding globulin, and elevation in free estradiol levels [76]. Several studies reported that women with obesity in post‐menopause may have an increased risk of BC due to an increased production of estrogen hormone from adipose tissue [58], as well as an increased local production of this hormone, due to the conversion of androgens via aromatase [77]. Obesity is characterized by a chronic low‐grade inflammation that it is known to play a role in promoting BC. Indeed, elevated levels of C‐reactive protein, an indicator of active inflammation, have been associated with an increased risk of BC in women with obesity [78]. Kolb et al. identified obesity‐associated NLRC4 inflammasome activation coupled with IL‐1 signaling as promoter of BC progression [79]. Moreover, it has been documented the participation of macrophages in obesity‐associated angiogenesis in humanized mammary adipose tissue through CCL2 and IL‐1β [80].

#### 3.2.2 Extracellular Vesicles

Recently, in addition to soluble mediators, extracellular vesicles (EVs), nanometer‐sized particles enclosed within a phospholipid bilayer membrane, are emerging as new players in obesity/BC communication [81, 82, 83, 84, 85]. EVs cargo is not only composed of several common proteins, such as Rab GTPases, annexin, tetraspanins (CD9, CD63, and CD81), integrins, adhesion molecules, heat shock proteins, matrix metalloproteinases, and MHC‐I and ‐II, but also includes specific cargo including nucleic acids that reveal the exclusive molecular signature of the releasing cells [86]. Several reports highlight the role of tumor‐derived EVs in modulating many aspects of BC cell biology [85, 87]. In the context of obesity, it has been demonstrated that leptin increases the release of EVs by epithelial mammary carcinoma cells. This occurs at the post‐transcriptional level through an upregulation of Tsg101 expression, which involves the activity of the chaperone protein Hsp90 [88]. Moreover, analysis of the proteomic profile of these leptin‐induced EVs revealed a significant enrichment in biological processes, molecular functions, and cellular components mainly associated with mitochondrial structures and activity [89]. Nowadays, it is well known that adipose tissue secretes high amounts of EVs and many authors investigated the role of adipose tissue released EVs in influencing BC biology. EVs from adipose tissue explants obtained from subjects with obesity increased growth of MCF‐7 and invasive properties of MDA‐MB‐231 BC cells via ERK/MAPK and PI3K/AKT pathways, respectively [90]. In addition, adipocyte‐EVs can support tumor growth, progression, and metastatic capacity of BC cells “in vitro” and “in vivo”, increasing the expression and function of the transcription factor HIF‐1α [84]. The most studied components of EVs cargo are miRNAs, small noncoding RNAs involved in the post‐transcriptional regulation of gene expression. Interestingly, analysis of circulating EV‐miRNAs in patients with BC with different BMI revealed the down‐regulation of let‐7a content in EVs from patients with overweight/obesity affected by BC compared with patients with normal weight. Of note, low levels of let‐7a were associated with higher grade and a reduced survival of patients with BC [91]. Thus, EV‐miRNAs may reflect a different profile with respect to BMI in women with BC. It has also been reported that EVs from breast adipose tissue of women with obesity are able to reprogram BC cells altering tumor cell metabolism. This may be due to an upregulation of obesity‐associated EV‐miRNAs, such as miR‐155‐5p, miR‐10a‐3p, and miR‐30a‐3p, which sustain an increase in cell proliferation and mitochondrial activity in BC cells [92].

## 4 Obesity and BRCA 1 and BRCA 2 Mutation Carriers

It is now well recognized that breast carcinoma is not merely a genetic disease, but local and systemic alterations associated with important host factors, such as obesity, could complement genetic modifications and tumor microenvironment effects in the promotion of cancer.

The association of several metabolic and hormonal factors with BC risk in the general population and in *BRCA* mutation carriers has been largely described. Particularly, it has been proposed that exposure to these factors may potentially modulate *BRCA* penetrance. However, evidence from studies to date is incomplete and conflicting. Knowledge on the role of nongenetic risk factors for BC development in *BRCA* mutation carriers is important for providing evidence‐based guidance to design risk‐adapted prevention strategies.

### 4.1 Impact of Obesity in Women Affected by BRCA‐Positive Breast Cancer

To date, several case–control and cohort studies have been conducted to investigate the impact of obesity and change in body size on samples of women with *BRCA1* and *BRCA2* mutations (Table 1), with conflicting results. Some reports have found that preserving a lower body weight or weight loss in young adulthood protects against early‐onset BRCA‐associated BC [93, 94]. Indeed, it has been reported that healthy weight at menarche and at age 21 was significantly associated with delayed age at BC onset in 104 *BRCA1* or *BRCA2* mutation carriers [93]. In a multicenter study, which included a total of 1073 pairs of women carrying a harmful mutation in either *BRCA1* (*n* = 797 pairs) or *BRCA2* (*n* = 276 pairs), Kotsopoulos et al. showed that weight loss of at least 4.5 kg between the ages 18 and 30 was associated with a significant reduction in BC risk (34%), while change in body weight at an older age did not influence BC risk [94]. Conversely, Chang‐Claude et al. provided evidence that BMI had no effect on age at onset for BC among 419 *BRCA1* mutation carriers [95]. Other studies have reported that increased fat mass or elevated body weight in adulthood was significantly associated with increased cancer risk [96, 97, 98]. In a multicenter longitudinal cohort study, Kim et al. found no association between BMI and BC risk in 3734 *BRCA* mutation carriers [99]. Accordingly, data from a large international study of women with a *BRCA1/2* mutation, analyzed by different Mendelian randomization approaches, indicated that a higher BMI was associated with lower risk of premenopausal BC [100]. In a retrospective cohort study, including 719 *BRCA1/2* mutation carriers, no association was found between body weight and premenopausal BC, while overweight and weight gain were reported to rise postmenopausal BC risk [101]. More recently, using a pooled cohort of 8091 *BRCA1/2* variant carriers, Kast et al evaluated the relationship of BMI and weight changes with pre‐ and postmenopausal BC risk. The data indicated, in a retrospective analysis, that in premenopausal women higher young‐adult BMI is associated with lower BC risk in both *BRCA1* and *BRCA2* variant carriers, while no association was found in postmenopausal patients. In a prospective analysis using data from *BRCA1* carriers, higher BMI and adult weight gain were associated with higher risk of postmenopausal BC, suggesting that weight maintenance is important in postmenopausal women with mutations [102].

**TABLE 1:** Studies showing correlation between body size and BRCA‐associated breast cancer.

Study design	Population	Sample size	Findings	Ref.
Case‐series study	*BRCA1/2* mutation carriers	*104*	Normal weight (vs. overweight) at menarche and at age 21 were significantly associated with older age of breast cancer onset among women with *BRCA1* or *BRCA2* mutations.	[93]
Case–control study	*BRCA1/2* mutation carriers	*1073*	Weight loss in early adult life (age 18 to 30) was associated with a significant reduction in breast cancer risk.	[94]
Cross‐sectional study	*BRCA1* mutation carriers	*46*	BMI had no effect on age at onset for breast cancer.	[95]
Randomized controlled study	*BRCA1/2* mutation carriers	*502*	Higher fat mass was significantly associated with BRCA‐related cancer, with greater effect in BRCA2‐positive women.	[96]
Case–control study	*BRCA1/2* mutation carriers	*137*	After adjustment for age, maximum lifetime BMI and physical activity, a positive association was found between total energy intake and BRCA‐related breast cancer risk. Age at the time the subjects reached maximum BMI was significantly related to an elevated breast cancer risk. A direct and significant relationship was noted between maximum weight gain since both age 18 and 30 years and breast cancer risk.	[97]
Case–control study	*BRCA1/2* mutation carriers	*200*	Breast cancer risk was significantly increased in over‐weighted and obese women.	[98]
Longitudinal cohort study	*BRCA1/2* mutation carriers	*3734*	Women with BMI at age 18 ≥ 22.1 kg/m^2^ had a decreased risk of developing post‐menopausal breast cancer compared with women with a BMI at age 18 between 18.8 and 20.3 kg/m^2^. BMI at age 18 was not associated with risk of pre‐menopausal breast cancer.	[99]
Randomized study	*BRCA1/2* mutation carriers	*22,588*	A higher BMI is associated with lower risk of premenopausal breast cancer, but was not statistically significantly associated with postmenopausal breast cancer.	[100]
Retrospective cohort study	*BRCA1/2* mutation carriers	*719*	No association between body weight and premenopausal breast cancer was observed, while overweight and weight gain increased postmenopausal breast cancer risk.	[101]
Prospective cohort study	*BRCA1/2* mutation carriers	*8091*	Higher young‐adult BMI was associated with lower premenopausal breast cancer in the retrospective analysis. In the prospective analysis, higher BMI and adult weight gain were associated with higher risk of postmenopausal breast cancer only for *BRCA1* carriers.	[102]

In recent years, research exploring the potential impact of other lifestyle factors, such as diet and physical activity, on the risk of BC in individuals with a *BRCA* mutation has also emerged. The Mediterranean diet, characterized by high consumption of fruits, vegetables, legumes, olive oil, and fish, has been linked to a lower risk of BC due to its anti‐inflammatory and antioxidant properties, as well as its potential effects on hormone levels and insulin sensitivity. Randomized controlled trials demonstrated that a Mediterranean dietary intervention substantially improves metabolic and anthropometric parameters that represent potential modulators of *BRCA* penetrance [96, 103, 104, 105]. Moreover, several studies report a protective role of physical activity against BC among *BRCA* mutation carriers, especially during young or early adulthood [106, 107, 108]. Although these studies provide valuable insights into the possible benefits of weight control, dietary patterns, and physical activity in reducing BC risk and improving outcomes in genetically predisposed individuals, further studies are required to provide more robust evidence about the complex interplay between lifestyle factors and BC risk among *BRCA* mutation carriers.

### 4.2 Molecular Mechanisms Linking Obesity and BRCA Penetrance

The molecular mechanisms underlying a link between obesity and BC development in *BRCA1/2* mutation carriers are still under investigation due to the multifaceted nature of obesity and the diverse oncogenic alterations that can drive BC molecular subtypes. It has been suggested that factors and pathways associated with obesity, such as dysregulation of hormone biosynthesis and adipokine balance, abnormalities of the IGF‐I system and signaling, and increased production of inflammatory mediators may affect BC penetrance in women with *BRCA1* or *BRCA2* mutations by contributing to DNA damage, promoting cell proliferation, and interacting with hormone receptors. Previous “in vitro” studies revealed that exposure to estrogen, along with its subsequent metabolism, in cells lacking the BRCA1 gene has been shown to result in an increased incidence of genomic instability, an established hallmark of early BC development. Particularly, it has been demonstrated that treatment with estrogen and estrogen metabolites can cause DNA damage in *BRCA1* heterozygous breast epithelial cells. Moreover, BRCA1 exerts a regulatory function in the estrogen metabolism and the resulting DNA damage by down‐regulating the expression of estrogen‐metabolizing enzymes, such as CYP1A1 [109]. Estrogen stimulation positively modulates expression of BRCA1 at the mRNA and protein level. On the other hand, BRCA1 is able to induce ERα mRNA expression and decrease ERα signaling [110].

Epidemiological observations reported the efficacy of salpingo‐oophorectomy, which significantly reduces estrogen levels, in decreasing the risk of BC in *BRCA* mutation carriers [111]. Additionally, the use of selective estrogen receptor modulators like tamoxifen and aromatase inhibitors has been investigated as a preventive measure for *BRCA* mutation carriers. Pharmacologic therapy with these agents is associated with a significant risk reduction in both *BRCA1* and *BRCA2* mutation carriers [112, 113, 114]. Adipocytes strongly express the enzyme aromatase, the product of the CYP19 gene that catalyzes estrogen biosynthesis. It has been shown, in a prospective cohort study of 141 women with germline *BRCA1* and *BRCA2* mutations, that elevated BMI was associated with an enhanced expression of P450 aromatase in both pre‐menopausal and post‐menopausal women. Moreover, the adipokine leptin, a well‐known biomarker of adiposity and a positive modulator of aromatase expression [68], positively correlated with both BMI and aromatase expression in women with either *BRCA1* or *BRCA2* mutations [115]. The available data collectively indicate that excess adiposity may enhance local estrogen production, which could subsequently promote tumor growth in individuals with DNA repair insufficiency resulting from germline mutations in the *BRCA1* or *BRCA2* genes.

Several studies have established that obesity promotes chronic low‐grade inflammation, particularly in white adipose tissue, which leads to increased production of chemokines and inflammatory cytokines [116]. In women who have either a mutation in the *BRCA1* or *BRCA2* genes, there was a positive correlation between circulating C‐reactive protein, IL‐6, and leptin and both BMI and aromatase levels, while a negative association was observed for adiponectin and sex hormone‐binding globulin [115].

Other lines of evidence indicate that dysregulation of the IGF‐1 system may represent an additional mechanism linking obesity with *BRCA* mutant carriers. A mechanistic study by Maor et al. [117] showed that IGF1 increases *BRCA1* gene expression and enhances *BRCA1* promoter activity, suggesting a functional interaction between the BRCA1 and IGF‐I systems relevant to BC biology. In primary breast tumors from patients carrying *BRCA1* mutations, it has been demonstrated that there is a significant increase in IGF‐I receptor levels compared with tumors from sporadic (nonfamilial) patients with BC [118]. In addition, IGF‐I protein expression was markedly enhanced in tumors of *BRCA1* mutation carriers in comparison with matched sporadic tumors [119]. Moreover, a case–control study of high genetic‐risk women revealed a higher penetrance of BC in those with elevated IGF‐I levels [120]. Recently, an elegant study by Brown and colleagues has provided mechanistic evidence that further elucidates the link between elevated BMI and BC development in *BRCA* mutation carriers. Using tissue microarrays from noncancerous breast tissue obtained from women with *BRCA* mutations, the authors found a positive correlation between BMI and epithelial cell DNA damage. RNA‐sequencing data revealed significant metabolic dysfunction within the breast adipose microenvironment of individuals carrying a *BRCA* mutation. This dysfunction includes the activation of estrogen biosynthesis, which exerts a significant impact on adjacent breast epithelial cells. Indeed, the study demonstrated that the inhibition of estrogen biosynthesis or estrogen receptor activity can lead to a reduction in DNA damage in breast epithelial cells that are heterozygous for the *BRCA* mutation. Moreover, the authors demonstrated additional obesity‐associated factors, such as leptin and insulin, as possible drivers of DNA damage. Treatment with metformin, leptin neutralizing antibodies, and PI3K inhibitor reduces damage induced by the obese breast microenvironment. Finally, in *BRCA1* heterozygous knockout mouse models of diet‐induced obesity, it has been clearly evidenced that increased adiposity is associated with mammary gland DNA damage and an augmented penetrance of mammary tumors [121].

## 5 Conclusions

Women with confirmed BC‐linked genetic variants, such as *BRCA1*/*2* mutations, require accurate cancer risk estimation and appropriate guidance for risk reduction. The National Comprehensive Cancer Network (NCCN) Guidelines for Genetic/Familial High‐Risk Assessment in Breast Cancer [122] highlight the most appropriate management strategies in women with these pathogenic or likely pathogenic variants.

The growing prevalence of obesity, coupled with its established link to an increased risk of BC, underscores the urgent need for a deeper understanding of how these factors intersect, particularly in individuals with *BRCA1/2* mutations. While *BRCA1/2* mutations are well‐documented as high‐penetrance risk factors for BC, the influence of obesity on cancer progression in these mutation carriers remains inadequately addressed and often unclear.

Several epidemiological studies suggest that obesity and changes in body weight may modulate *BRCA* penetrance. However, the available data remain conflicting, and the exact impact of obesity on BRCA‐related BC is partially explored. Potential mechanisms linking obesity with BC progression in *BRCA* mutation carriers include chronic inflammation, altered adipokine levels, dysregulated hormone signaling, and insulin/growth factor pathways, all of which may contribute to DNA damage, promote cell proliferation, and interact with hormone receptor activities. Among the mechanisms involved in the intricate connection between obesity and BC, recently growing evidence revealed a role for obesity related‐EVs. Up to now no data concerning the impact of EVs on the risk and biology of BC in *BRCA* mutation carriers are available. Thus, this issue deserves more investigation, since it may allow to determine additional early biomarkers and putative therapeutic target for *BRCA* mutation carriers. Overall, a more thorough understanding of the complex interaction between obesity and *BRCA* mutations is essential for developing personalized clinical management strategies that can reduce BC risk in this population through lifestyle modifications and early detection. However, additional research is urgently needed to better define the role and impact of obesity in BC biology in *BRCA* mutation carriers, with the goal of providing more targeted and effective treatments and ultimately improving the quality of life and outcomes for women at increased genetic risk for BC.

## Conflicts of Interest

The authors declare no conflicts of interest.
